# Hemodynamic Management with Vasopressin for Cardiovascular Surgery

**DOI:** 10.3390/medicina60122064

**Published:** 2024-12-16

**Authors:** Hideyuki Kato, Bryan J. Mathis, Tomonari Shimoda, Tomomi Nakajima, Chiho Tokunaga, Yuji Hiramatsu

**Affiliations:** Department of Cardiovascular Surgery, University of Tsukuba Institute of Medicine, Tsukuba 305-8575, Japan

**Keywords:** vasopressin, vasoplegic, cardiovascular, arginine vasopressin, epinephrine, norepinephrine

## Abstract

*Background and Objectives*: Vasopressin increases blood pressure through aquaporin-2-mediated water retention and is useful for managing hemodynamics after surgery. However, even after decades of study, clear clinical guidelines on doses and ideal use cases after cardiovascular surgery remain unclear. Here, the existing literature is synthesized on vasopressin use for cardiac surgeries and coupled with real-world clinical experience to outline a clearer clinical path for vasopressin use. *Materials and Methods*: Literature from 1966 to the present was searched, and information on surgical outcomes for cardiovascular surgery was extracted. Clinicians from the University of Tsukuba with extensive vasopressin experience in pediatric cardiovascular patients were consulted for general use guidelines. *Results*: Vasopressin response after cardiovascular surgery is multifaceted, and low-power trials, plus conflicting study reports, generally render it as a secondary choice behind norepinephrine. Clinical experience indicates that low doses of 0.2–0.3 mU/kg/min with constant blood pressure and oxygen monitoring for response are required. Although sole use is not recommended, vasopressin may aid in controlling hemodynamics when given with other volemic or osmolal drugs. *Conclusions*: Vasopressin may work in a select population of first-line non-responders, but relevant response factors remain unanalyzed and clear guidelines for use remain unestablished. Future, large-scale studies are needed to delineate temporal and demographic characteristics that affect response to vasopressin for the purpose of managing post-surgical capillary leakage and hemodynamics.

## 1. Introduction

Vasopressin, first identified by Oliver and Schäfer in the late 19th century, has become integral to clinical practice and is leveraged for multiple uses in behavioral, neurological, cardiac, and emergency medicine due to its role as a mediator of blood pressure homeostasis [1]. Recently, it has garnered interest and investigation as a treatment for septic shock, but administration to manage hemodynamics after cardiovascular surgery represents an additional frontier.

### Vasopressor Use During Cardiovascular Surgery

Cardiovascular surgery is invasive and results in hemodynamic changes (i.e., fluctuations, usually drops, in blood pressure) outside of the body’s homeostatic ability, resulting in potential organ and tissue ischemia due to low cardiac output and hypoperfusion. Additionally, ischemia is associated with many severe cardiovascular conditions, such as myocardial infarctions, septic shock, or pulmonary embolisms that affect circulation of blood. As sudden restoration of oxygen after extended ischemia can cause reperfusion injury, external hemodynamic support in the form of cardiopulmonary bypass (CPB) or extracorporeal membrane oxygenation (ECMO) is used in the surgical suite to maintain steady blood oxygen and circulation (including pressure) during surgery. CPB creates a pressurized circuit attached to the vascular system that compensates for an intentionally stopped heart during surgery, while ECMO is a pump assistance system that increases blood flow but does not support the heart directly.

These technologies themselves, however, carry risks during use, such as reduced perfusion, arrhythmias, and arterial pressure drops, as well as weaning issues (post-surgical bleeding) from the heparin anti-clotting drugs used to initiate external circulation, mechanical damage to erythrocytes due to the pump/tubing interface, and electrolyte disturbances. Of particular concern during external circulation is hypotension, as a severe drop in blood pressure/vascular resistance overwhelms the autonomic regulation of the heart, and cardiac arrest/cardiogenic shock may occur [2]. Vasoplegic shock, where vascular resistance drops sharply after CPB, is a serious complication, and pressure must be quickly restored to prevent cardiac arrest [3]. It is for the treatment of hypotension and cardiogenic/vasoplegic shock due to mechanical circulatory intervention that vasopressors or inotropes such as norepinephrine or vasopressin are used in both emergency and corrective cardiovascular surgery.

## 2. Materials and Methods

PubMed was searched for all manuscripts related to vasopressin or its analogues (e.g., desmopressin and felypressin) in the English language and from January 1983 to 2023, later extended to July 2024. Human studies and reports were analyzed for results based on the use of vasopressin or its clinical analogues in cardiovascular surgery and recovery from such surgery.

## 3. Physiological and Clinical Parameters of Vasopressin

### 3.1. Vasopressin Generation, Regulation, and Molecular Action

**Figure 1:**
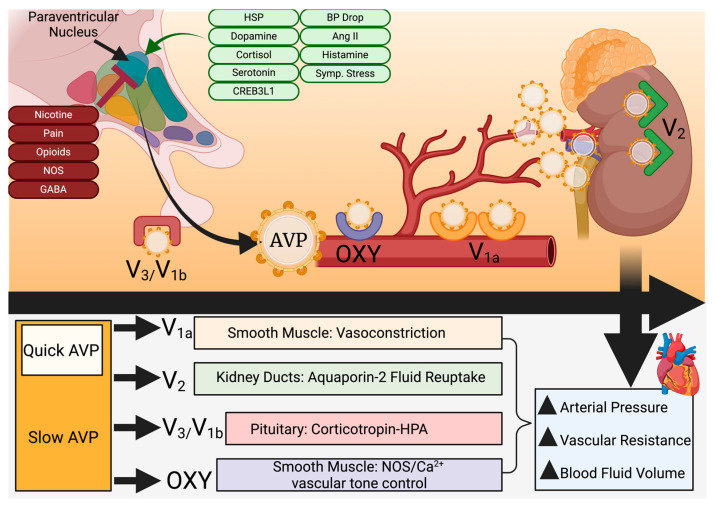
Vasopressin secretion, targets, and action. Vasopressin is released on demand from the paraventricular nucleus within the hypothalamus through the pituitary gland. Roughly 10–20% is released directly into the bloodstream, while the rest is systemically released under the control of feedback mechanisms and external stimuli. Four G-protein-coupled receptors, located in smooth muscle (OXY/V1a), the pituitary gland (V1b/V3), and the kidney collection ducts (V2), respond to vasopressin by vasoconstriction, reabsorption of water, or vascular tone control through multiple mechanisms. This collectively raises vascular resistance and blood fluid volumes, increasing arterial pressure. Ang II = angiotensin II; AVP = arginine vasopressin; BP = blood pressure; CREB3L1 = CAMP responsive element binding protein 3-like 1; GABA = *γ*-aminobutyric acid; HPA = hypothalamic-pituitary-adrenal axis; HSP = heat shock protein; NOS = nitric oxide species; OXY = oxytocin-like receptor. Created at biorender.com.

Secreted by the hypothalamus over a 1–2 h synthesis cycle to maintain osmotic/blood pressure and mineral homeostasis, the cyclic nonapeptide 8-arginine vasopressin (AVP) is generated by selective magnocellular cells and enters the systemic circulation from posterior pituitary secretion upon stimulation by increased plasma osmolarity and lower blood pressure [1,4,5,6,7]. Upon activation, 10–20% is dumped into systemic circulation for rapid action while the remainder is released slowly over time based on stimulus feedback (Figure 1) [4].

AVP is similar in structure and function to oxytocin, differing in only amino acid positions 3 and 8 [8]. It is initially synthesized as a large zymogen and selectively cleaved by endoprotease PC1/3 (encoded by the PCSK1 gene; previously known as convertase SPC3) to generate neurophysin II (a transporter), copeptin (a glycoprotein useful as a biomarker of endogenous vasopressin levels), and active AVP [9,10,11]. PC1/3, a member of the family of subtilisin-like PCKS enzymes that are also secreted as zymogens, cleaves numerous other prohormones, including oxytocin, which is generated in equimolar amounts as prooxytocin [12]. The specificity and rate of PC1/3 with regard to AVP are dependent on the size of the carboxy terminus of PC1/3 as well as self-association with variably sized species of PC1/3 itself that circulate within the cellular microenvironment [10,12]. Thus, post-translational regulation of AVP by the rate-limiting enzymatic cleavage of provasopressin serves as the primary endogenous regulator in heart failure or other vascular conditions with volumetric or edematous involvement [9]. In addition to volumetric stimuli and PC1/3, regulation of vasopressin release is accomplished through multiple molecules, including glucocorticoids (e.g., cortisol), mineralocorticoids, cAMP-responsive element binding protein 3-like-1 (CREB3L1), estrogen, serotonin, dopamine, histamine, angiotensin II, and the heat-shock response [4,13]. Clinical and somatic stimuli that increase AVP release are pain, nicotine use, and nausea, while nitric oxide, opioids, and GABA function to inhibit secretion (Figure 1) [4]. Healthy individuals have a plasma concentration of 4 pg/mL or less, with concentrations of up to 20 pg/mL in hyperosmolar blood [4].

Vasopressin is metabolized by a zinc-dependent cystinyl aminopeptidase (vasopressinase), which resides in the kidneys and liver to cleave vasopressin and similarly structured, short-peptide hormones (i.e., oxytocin and bradykinin), but cannot easily act on synthetic analogues such as desmopressin [14,15]. While there is no current evidence that oxytocin or vasopressin share feedback control mechanisms, a relatively short half-life for AVP of 10–35 min (similar to oxytocin) and the presence of constitutively active vasopressinases in the liver and kidneys may have relieved selective pressure to evolve direct feedback inhibition of AVP [16,17].

An entire class of specialized G-protein-coupled receptors (V1a, V2, and V3/V1b) for AVP are distributed in the spleen, liver, kidneys, and smooth muscle of the vascular system (V1a), with V2 receptors concentrated in the renal collection duct to modulate water permeability through aquaporin-2 [18,19]. The V3/V1b receptors are located within the pituitary gland and regulate the hypothalamic–pituitary–adrenal axis through modulation of corticotropin release [20]. Other smooth muscle-resident receptors, known as oxytocin-type, can also bind to vasopressin (due to structural similarity) and function through calcium flux and endogenous nitric oxide action to additionally promote respective vasoconstriction or dilation (Figure 1) [17,19,21].

### 3.2. Physiological Activity, Clinical Utility, and Monitoring of Vasopressin

Unlike synthetic desmopressin, vasopressin has broad receptor affinity, high vasoconstrictive activity, and a shorter plasma half-life of around 10–35 min (versus approximately 158 min for desmopressin), which allows for corrective maintenance of blood pressure during hemorrhaging or blood loss [17,22]. Clinically, when catecholamine must be reduced to spare cardiac oxygen and reduce atrial fibrillation, or if catecholamine non-response is encountered, control over blood pressure and solute concentration in the clinical setting can be targeted through vasopressin or its synthetic derivatives (e.g., pro-drugs terlipressin/glypressin or desmopressin) [17,23]. Each derivative molecule is engineered for effect and half-life to treat septic shock, cardiac arrest, hemorrhage, and other volemic issues, but most hospitals will select one or two only based on clinical use and experience [17]. The pharmacology, structure, and specific use cases for each derivative are reviewed in detail elsewhere [24]. Regardless of choice, postoperative manipulation of blood pressure and water balance with vasopressors can result in hyponatremia and dehydration as common negative side effects [25,26]. Vasopressin also functions as a key mechanism in buffering plasma osmolality, responding to even a 2% decrease in body water loss with V2 receptor binding to increase water volume to tightly maintain solute concentrations within a 275–290 mOsm/kg range [17]. There is also evidence that von Willebrand factor and other clotting molecules are upregulated by the action of V2 receptors, making it potentially advantageous when primary and secondary hemostasis mechanisms are attenuated due to coagulopathy [17]. It may be especially useful for patients who undergo cardiopulmonary bypass during cardiac operations that may negatively affect platelet activity [27].

While somatic effects are easily monitored, vasopressin itself is unstable when sampled from the blood, and, as it primarily binds to platelets, incomplete platelet removal will result in false readings [28]. For this reason, copeptin, a precursor molecule released in a 1:1 ratio with AVP, has been proposed as a more serum-stable and reliable clinical measure of AVP levels in the systemic circulation [28]. Yamashita and colleagues found that higher copeptin levels were associated with increased risk of acute kidney injury, indicating that higher levels of vasopressin were circulating. Conversely, Colson and colleagues reported that high preoperative copeptin levels were associated with increased risk of vasoplegia, indicating less endogenous vasopressin available after the surgery to generate sufficient vasoconstriction [29]. Thus, copeptin, as a representative of vasopressin, becomes a molecule of increased clinical importance when managing hemodynamics.

## 4. Operative Use of Vasopressin for Cardiac Surgery Recovery

### 4.1. Intraoperative Use of Vasopressin During Cardiac Surgery

Cardiac surgeries may lead to postoperative low cardiac output syndrome, vasoplegic syndrome (VPS), major blood loss, coagulation cascade derangement, and arrhythmias [27,30]. The use of fluids, inotropes, and catecholamines in response may further exacerbate potential conditions by inducing arrhythmias [31]. In these cases, the hemodynamic affinity of vasopressin may be useful in restoring homeostasis after surgical disruption of arterial pressure. Some studies have reported on the benefits of intraoperative vasopressin use for this purpose during cardiac operations, mainly through prevention of VPS occurrence and better preservation of myocardial contractility [32,33,34,35]. This was seen in a study by Elgebaly and colleagues, which demonstrated that intraoperative administration of 0.03 IU/min vasopressin for on-pump coronary artery bypass graft (CABG) patients with systolic dysfunction led to increased left ventricular function during separation from cardiopulmonary bypass, as well as a reduction in catecholamine use [32]. In addition to preload and afterload changes, vasopressin is associated with improved cardiac contractility [36,37]. It accomplishes this by elevating intramyocardial calcium concentration and coronary artery vasodilation due to increased systolic pressure [38]. In another study, vasopressin was associated with decreased central venous pressure as well as less pronounced cerebral circulation constriction [4,39]. Furthermore, intraoperative use of vasopressin during hypotensive periods for CABG patients has also been associated with reduced catecholamine use [34]. Pulmonary arterial pressure increases were not observed with the administration of vasopressin in several studies, demonstrating its intraoperative safety [32,34,40].

Of note, there are scarce studies featuring vasopressin usage in pediatric cardiac shock patients, as adults tend to receive vasopressors for surgeries to repair damage (e.g., heart disease) while the majority of pediatric patients receive surgeries for congenital malformation repair (e.g., tetralogy of Fallot). A 2021 meta-analysis by Farias and colleagues analyzed only six studies with 160 total patients for vasopressin use to stabilize hemodynamics 2 h after repair surgeries and found significant increases in blood pressure and lowered heart rates [41]. Further analysis of eight studies with 338 total patients found lower lactate levels, central venous pressure, and fluid balance 24 h after vasopressin [41]. With regard to safety, analyses have found no significant differences in adverse events between adults and children with regard to vasopressin vs. other vasopressors [42]. Therefore, while vasopressin does seem safe and to have some beneficial effect in hemodynamic management after pediatric cardiac surgeries, larger studies are needed to delineate the full benefits and drawbacks of vasopressin in these younger patients.

### 4.2. Specific Use Case: The Role of Vasopressin for Vasoplegic Syndrome After Cardiac Surgery

VPS, or vasodilatory shock, is a well-recognized complication after cardiopulmonary bypass [43]. The reported incidence varies from 5% to 44% and carries significant morbidity and mortality risks [44,45]. It is characterized by profound systemic hypotension or low mean arterial pressure due to persistently low systemic vascular resistance (SVR) associated with normal or supra-normal cardiac functions, but exact thresholds for cardiac parameters vary throughout the literature. In general, however, systemic hypotension is present (e.g., mean arterial pressure [MAP] < 65 mmHg), an increased cardiac index is often (but not always) seen (e.g., >2.2 L/min/m^2^), and low systemic vascular resistance (e.g., <800 dynes·s/cm^5^) with normal or increased filling pressures is usually encountered [3]. Low MAP is universally seen due to decreases in vasomotor tone after non-response to escalating doses of catecholamines. Additionally, higher lactate levels will indicate a more severe status, as lactate is released from skeletal muscle and usually cleared by the liver and kidneys unless affected by low blood pressure. In such cases, monitoring of lactate could be an important indicator of response to vasopressin titration.

VPS after cardiovascular surgery is multifactorial but putatively related to the release of proinflammatory molecules that mediate the drop in blood-borne vasoactive mediators. Importantly, vasopressin plays a crucial role in preventing VPS as a non-catecholamine vasopressor by diminishing nitric oxide (NO) production, as V1a (AVPR1A) is a key player in mediating vasoconstriction independently of catecholamines. V1a inhibits KATP channel opening, thereby reducing NO production and offering a catecholamine-independent mechanism for inducing vasodilation. The administration of vasopressin thus proves beneficial in VPS by inactivating KATP channels, suppressing NO synthesis through V1a receptor binding, and mitigating the NO-induced increase in cGMP [3]. Furthermore, V1a activation leads to a protein kinase C-dependent elevation in intracellular calcium, achieved both directly through Ca++ channel opening and indirectly by inhibiting KATP channels. This process, coupled with PKC activation, inhibits MLC phosphatase, decreases iNOS expression, ablates NO formation in response to inflammatory cytokines, encourages the release of vasoconstrictors (such as endothelin-1 and thromboxane A2), and ultimately enhances vascular catecholamine sensitivity [3].

This concordant opposition of NO and vasopressin may be challenging for surgeons, as vasopressin in patients receiving NO to treat pulmonary hypertension may be theoretically harmful. However, a report in neonates demonstrated that concomitant vasopressin and NO improved blood oxygenation and mean arterial pressure within 1 h after vasopressin was started, while a clinical report from Currigan and colleagues revealed that vasopressin does not constrict pulmonary arteries or increase pulmonary vascular resistance, unlike norepinephrine or epinephrine, making it useful when NO is used to ameliorate pulmonary hypertension [46,47]. Vasopressin retains additional utility for surgeons using inhaled NO in that this pulmonary-sparing effect relieves the right ventricle of increased load yet delivers enhanced oxygenation to the tissue [48]. As the increased systemic diastole also increases output (as cardiac perfusion mainly occurs during diastole), this reliable myocardial oxygenation could be a welcome effect in maintaining cardiac output after surgery.

### 4.3. Vasopressin and Fluid Balance

In spite of its utility, vigilant monitoring of water balance before and during administration is critical for vasopressin use, as it has a negative effect on urine output. This is evidenced by its central role in edematous diseases (e.g., cirrhotic liver or congestive heart failures), where elevated AVP levels force water retention, increase blood pressure, and worsen the edema [49]. This is due to the potency of vasopressin, which causes maximum urine solute density at 5–7 pg/mL concentrations in the plasma, close to the 4 pg/mL reported under normal conditions [4]. Levels may be elevated in volemic diseases, as seen in clinical reports where congestive heart failure patients had mean vasopressin concentrations of 9.5 pg/mL, twice the normal value, in one study and mean values of 3.5 pg/mL versus 2.0 pg/mL in controls for another study [50,51]. Meanwhile, liver cirrhosis studies frequently reported hyponatremia, but a study of 19 liver cirrhosis patients found lower levels of vasopressin after increasing blood volume and osmolality via saline administration, indicating an active regulation of vasopressin levels in response to osmolality. Within this population, a subgroup with renal dysfunction retained higher levels of vasopressin [52]. Thus, diverse reports link AVP with water retention and high blood pressure during the course of chronic, edematous diseases, and elevated endogenous levels may complicate management of post-surgical hemodynamics with exogenous vasopressin in patients with both VPS and pre-existing renal/hepatic conditions.

Endogenous AVP after surgery has long been studied with regard to fluid output and concentration. In 1985, Fieldman and colleagues reported lower urine output after abdominal surgery, while return of AVP from a post-surgical peak to nominal concentrations resulted in normal water excretion but with a lag period of hypertonic osmolar load until excreted solutes normalized [53]. However, this effect was probably based on kidney filtration rate and osmolar concentration, not AVP concentration [53]. A more recent study also found a post-surgical 6 h peak in AVP concentration that did not result in significant urine osmolality changes over a 6 h to 1 week post-surgical period [54]. Based on those results, the endogenous AVP elevation in response to surgery does not seem to strongly mediate urine concentration or renal function. Of note, Yamashita and colleagues reported that higher copeptin levels (reflective of higher endogenous vasopressin) after cardiac surgery did result in elevated risks of acute kidney injury [40]. In light of this evidence, endogenous AVP may not be a crucial molecule for restoring urinary homeostasis, and elevated levels might actually increase pressure on the kidneys after major surgeries. Thus, for hemodynamic management after cardiac surgery, exogenous vasopressin must be both short-term (hours to several days) and balanced to avoid VPS from too little and organ damage from too much combined endogenous and exogenous AVP.

Clinically, exogenous vasopressin after cardiac surgery has been reported to successfully manage hemodynamics with weak to insignificant effects on fluid output. This utility was reported by a 2018 meta-analysis of 625 total patients (n = 313), which found that vasopressin use reduced perioperative complication risks (OR 0.33, 95% CI 0.2–0.54), with the greatest reductions in vasodilatory shock and atrial fibrillation (new-onset only) and no changes in mortality risk [55]. This effect was also seen in a clinical report of 145 CPB patients in which vasopressin deficiency and angiotensin-converting enzyme (ACE) inhibitor use were deemed risk factors for vasodilatory shock [36]. Neither study reported excessive fluid output in response to vasopressin. Dünser and colleagues also reported vasopressin effectiveness in maintaining hemodynamics in postcardiotomy shock patients but did not report unusual urine concentrations or volumes [56]. However, the meta-analysis also found insignificant effects of vasopressin on postsurgical hyponatremia, indicating a trivial effect on water retention in cardiac surgery patients [55]. With regard to osmolarity effects by vasopressin in cardiac patients, a study by Yamashita and colleagues on 23 patients found insignificant changes in blood osmolarity even when copeptin levels (representative of vasopressin) were 8× higher (mean 20 pmol/L in the low group vs. mean 158 pmol/L in the high group); values in both groups remained in the mean 304–308 mOsm range [40]. A report by Jerath and colleagues on 85 pediatric, post-cardiac surgery patients who received vasopressin for advanced shock (up to 24 h median infusion time at 0.1 mU/kg/min per patient) found significant increases in urine output but no significant changes in urea; the increased urine effect was not seen in a separate non-cardiac group that received only a single infusion [57]. A similar effect was seen by Agrawal and colleagues, who found no changes in serum sodium concentrations during vasopressin infusion in pediatric cardiac surgery patients experiencing vasodilatory shock [58]. This was congruent to a trial conducted by Bigelow and colleagues, in which vasopressin after the Fontan procedure decreased chest tube drainage fluid but had no effect on sodium concentration, urine output, or kidney function [59]. Taken together, these data indicate that, while vasopressin may theoretically affect electrolyte balance and fluid, the clinical literature reports a more neutral role, making it suboptimal for active management of fluids.

### 4.4. Clinical Vasopressin Use and Cardiac Output

Although there is contrary evidence that vasopressin increases or decreases cardiac output, intensivists generally believe that it improves hemodynamics. Clinically, Wenzel and colleagues performed a randomized, double-blinded trial for out-of-hospital cardiac arrest featuring vasopressin versus epinephrine as a resuscitation drug. Vasopressin allowed more patients to reach hospital compared to epinephrine (29% vs. 20%, *p* = 0.02). Of those who reached hospital alive, 4.7% of AVP recipients were eventually discharged versus only 1.5% of epinephrine recipients [60]. At lower doses, vasopressin can maintain cardiac output, which may aid in resuscitation, but high-dose vasopressin has been reported in animal studies to reduce cardiac output in normoxic hearts while increasing myocardial function in hypoxic hearts [61]. A letter by Dünser and colleagues reinforced the contrary nature of vasopressin and cardiac output by reporting that high doses (20–30 U/h), sufficient to replace catecholamines and maintain MAP, were observed to reduce cardiac output [62]. Thus, vasopressin may be of limited utility for cardiac output.

The full mechanism of hemodynamic improvement by vasopressin remains unelucidated, but the main effect is vasoconstriction, through which increased peripheral vascular resistance increases systemic arterial pressure. This increased afterload commonly reduces cardiac output [63]. However, the clinical impression of vasopressin usage is that it sometimes improves hemodynamic instability. This may result from increased systolic and diastolic arterial pressure, which increases coronary artery flow to propagate and support robust cardiac function, especially in support of failing right ventricles. Even though vasopressin potentially has an influence on coronary perfusion, some studies report low-dose use without ischemic adverse events (0.3–0.5 mU/kg/min) in children with congenital heart disease who experienced CPB [64,65,66]. However, there is no current clinical trial to study correlations between coronary blood flow and vasopressin usage. Table 1 summarizes reported usage of AVP in clinical settings, with specific information on procedure, administration time, and patient numbers.

### 4.5. Starting Dosage

No currently reported, reliable biomarkers exist for prediction of response to vasopressin. Also, there are no concrete guidelines for vasopressin dosing, but published studies report ranges between 0.3 and 2 mU/kg/min. A pediatric study that successfully used vasopressin to manage hemodynamics in cardiac cases experiencing catecholamine-resistant vasodilatory shock reported starting doses of 0.5 to 3 mU/kg/min, with dosing adjusted by clinical course [58]. An international pediatric survey revealed that the majority of clinical vasopressin users reported ranges of 0.3 to 0.6 mU/kg/min as the most common [74]. Although higher initial doses may seem theoretically better, a study by Dubrawka and colleagues in 2021 analyzed doses equal to or less than 0.04 U/min (standard) versus doses higher than 0.04 U/min (“high”) for septic shock and found that the higher arterial pressures obtained came at the cost of lengthier shock duration and hospital stays (albeit with no differences in mortality) [75]. As seen in Table 1, low doses of 0.03 IU/min are frequently reported, with titration up until MAP stabilizes at around 65 mm Hg. The Dubrawka study thus indicates that low doses with individualized titration may be advisable for faster recovery. Monitoring of clinical parameters (e.g., SvO2, urine output, and blood pressure) while titrating will result in a better clinical outcome. Because of reduced cardiac output concerns at higher doses, lower starting doses with titration to the minimally effective dose seem to be prudent. 

Choosing to abruptly stop vasopressin versus titrating down, especially in non-responders, is a complex issue as there is currently no consensus as to how or when to stop. Because there will be at least some endogenous vasopressin remaining, titration is logical, but a large study (n = 1318) found that abrupt stoppage of vasopressin during combination norepinephrine was safe and beneficial for septic shock patients [76]. Titrating up may also be useful, as a double-blinded study in 50 CABG patients, where very-low-dose vasopressin was used prophylactically against VPS at 3 mU/min, found it to be effective [33]. A reduced need for norepinephrine was also observed, indicating that starting at minimal doses and titrating up based on clinical parameters/monitoring is viable in cardiac surgery patients as opposed to septic shock cases [33]. As for the discontinuation of concomitant vasopressors, a 2020 meta-analysis by Wu and colleagues found no real benefits to withdrawing vasopressin before concomitant norepinephrine with regard to mortality or length of stay, but larger studies are needed to explore the topic in detail [77]. 

Dosing may also be adjusted until response, but studies to date are unclear as to patient variables that directly influence response to vasopressin. A study of 938 ICU patients found that only intensive care location (surgical and neurosurgical) and lactate concentration (lactate is increased in any severe shock) were significant predictors of favorable response, which the authors also noted as evidence of catecholamine sparing [78]. Similarly, a septic shock report evaluating body mass effect on vasopressin utility found that body mass index (BMI) was not significantly correlated with AVP response if the dose was adjusted for BMI and weight [79]. Although vasoplegic shock also features higher lactate levels, similar studies on lactate levels with regard to vasopressin after cardiovascular surgery remain scarce. Only early response to vasopressin, evidenced by restoration of blood pressure during titration, has been found to be a reliable predictor [80]. Thus, current knowledge indicates that low, steady doses with room to titrate up are recommended for cardiac surgery, as body mass or demographics are not significant factors for response prediction.

Discontinuation is important, as long-term vasopressin use carries increased risks of liver and kidney damage via restriction of splanchnic flow and increases in beta and gamma subunits within sodium channels in the renal epithelium, possibly affecting electrolyte balance [81,82]. While long-term use (days to weeks) may cause issues, vasopressin is administered only during the acute phase of postoperative recovery (hours), and careful titration to the minimum effective dose ensures that exposure to acutely large or systemic, long-term boluses is rare. Therefore, long-term risks and exposure side effects are rare unless an individual patient has some distinctive anatomical issue that requires extended vasopressin administration.

### 4.6. Clinical Effect

Table 2 summarizes the extant reports on vasopressin dose and outcome parameters in cardiovascular surgery.

## 5. Clinical Use of Vasopressin Versus Norepinephrine

### 5.1. Norepinephrine/Noradrenaline

Norepinephrine (NOR) is a biogenic amine with a catechol center, synthesized by the adrenal medulla from a tyrosine precursor, and a member of a family of catecholamines (dopamine, norepinephrine, and adrenaline) that increase blood pressure by direct action on alpha-1 receptors in the smooth muscle of the vascular system as well as beta-1 receptors in the myocardium [83,84]. Stimulation of these receptors increases both vascular contractile (alpha-1, inotropic) and heart (beta-1, chronotropic) parameters, with dopamine having a beta effect in high doses and vasodilatory effects at low doses and NOR primarily having an alpha effect [85]. Compared to vasopressin at 1084.23 Da, norepinephrine is 170.19 Da, six times smaller, and has a much shorter half-life at 2 to 2.5 min [86]. Metabolized by monoamineoxidase and catechol-O-methyltransferase in the liver, norepinephrine must be constantly administered due to its short time of action [86]. Synthesis of these catecholamine neurotransmitters is regulated entirely by the rate-limiting step involving tyrosine hydroxylase and its multiple phosphorylation sites on four serine residues, while norepinephrine is created from dopamine by dopamine *β*-hydroxylase, and epinephrine is further derived from phenylethanolamine N-methyltransferase action upon norepinephrine [87]. The ability to rapidly generate a number of catecholamines from a single precursor (phenylalanine to tyrosine via phenylalanine hydroxylase) and then inhibit the rate-limiting synthesis enzyme via feedback inhibition offers the advantage of tight hemodynamic control. The rapid effect and short half-life make NOR a frequent first-line clinical choice [87].

### 5.2. Clinical Comparison: Norepinephrine vs. Vasopressin

In addition to first-line use in hemodynamic management, NOR has been used successfully as a second-line treatment for hepatorenal syndrome in midodrine and octreotide non-responders [88]. Compared to dopamine, a report of septic shock patients observed that NOR was significantly more effective at restoring heart rate, with significantly higher responders compared to dopamine (19/25, 76% vs. 10/25, 40%) [89]. However, another small trial of 53 septic shock patients reported the ability of selepressin to directly substitute for NOR at 2.5 ng/kg/min and give excellent performance in mean arterial pressure, days alive, and ventilation-free days [90].

In cardiovascular medicine, NOR, because of its focused alpha-1 action, is preferable to dopamine, which has demonstrated increased mortality in clinical studies of cardiogenic shock due to its strong beta-1 effect on the myocardium (higher heart rate and cardiac output with increased filling pressure) [85]. It has been proposed that, in cases where hemodynamic response is required, 0.1 μg/kg/min NOR has the same effect as 10 ng/kg/min angiotensin II; raising NOR to high doses (0.3 μg/kg/min) without increases in diastolic pressure (e.g., 20 mmHg within a few minutes) may be considered non-response [91]. In contrast, vasopressin was shown to be superior at resolving VPS with less atrial fibrillation after cardiac surgery in a direct comparison to NOR in the 2-year VANCS trial (n = 300) [31]. However, those data conflict with a study (n = 338) that directly compared NOR to vasopressin and reported no significant differences in primary outcomes after treatment for VPS, with vasopressin having significantly worse atrial fibrillation (NOR 11.83% vs. vasopressin 20.12%, *p* = 0.038) and ventricular arrhythmias (NOR 14.20% vs. vasopressin 24.85%, *p* = 0.014); this effect was attributed to the possibility that some vasopressin group patients also received NOR as part of a vasopressor management strategy and potential interference by milrinone and other inotropic agents [72]. Further conflicting evidence was reported in a recent meta-review of 1161 studies, totaling only 708 patients, that discovered no significant effects of vasopressin in treating VPS after cardiac surgery over other standard vasopressors (including NOR) but concluded that inconsistent methodologies, small study sizes, and heterogeneity in study populations complicate efforts to compare vasopressin with similar drugs [92].

Taken together, there is no concrete evidence that vasopressin can completely substitute for catecholamines in situations of complications after cardiac surgery. In fact, norepinephrine and vasopressin are often given concomitantly. When Vail and colleagues analyzed 584,421 patients with septic shock in 532 hospitals, they found that, of the 100,923 that received vasopressin, a mere 6.1% received only vasopressin while the rest were co-administered other vasopressors in up to 15 combinations [93]. Of these combination vasopressor patients (n = 142,110), 4.0% (n = 23,145) received both NOR and AVP [93]. Although no such data are currently available for cardiovascular surgery, the septic shock data indicate that AVP may be often relegated to second-line use after non-response to catecholamines or for combination use with NOR. While the expert consensus by the European Association of Cardiothoracic Anesthesiology and Intensive Care group carries recommendations on the use of both drugs, further studies are mandated to formulate criteria on selecting appropriate use cases [94]. A lack of standardization in dosing and study populations further complicates any effort to strongly recommend first-line or sole reliance on AVP over NOR, and use should be on a case-by-case basis.

## 6. Discussion

Clinical Analysis and Recommendations: The Surgeon’s Opinion.

What does this mean for the cardiac surgeon who faces hemodynamic challenges in patients, especially those with co-morbidities? What follows are comments from surgeons at our facility that reflect institutional experience and consensus from vasopressin use for cardiac surgery. At our institution, surgeons, intensivists, and anesthesiologists discuss the use of intravenous vasopressin as a team and make decisions based on (1) surgical progress (intra- or post-operative), (2) amount of pressure needed (e.g., if 10–15 mmHg pressure or more is required), (3) use of catecholamines (usually given first, with vasopressin as a second line for non-response or could be concomitantly given), (4) lactate status (high lactate indicates more severe shock, and lactate blood gas analysis can be used to monitor response), (5) volume loading (may be skipped entirely in some cases), and (6) general condition/anatomic considerations (e.g., presence of atherosclerosis). Volume loading, in particular, is usually difficult and of limited use, as cardiogenic shock features low cardiac output, coupled with high left ventricular end-diastolic pressure; extra volume will not increase output but may damage an already pressurized left ventricle. Thus, use of catecholamines and vasopressin may be preferable to fluid supplementation.

### 6.1. When to Use

1. To induce vasoconstriction/elevate blood pressure:

After cardiac surgery, patients often experience decreases in blood pressure attributed to low cardiac output, VPS, dehydration, deep sedation, etc. To normalize post-operative blood pressure and maintain adequate circulation, low-dose vasopressin administration is often effective in concert with compensatory treatments. However, there are responders and non-responders; it works beautifully for some patients but little for others, being a double-edged sword, as high doses may cause excessive vasoconstriction that increases cardiac afterload and leads to organ damage. The definition of non-response may vary by institution, but in general, there may be no increases in diastolic pressure over 10 to 20 min. Therefore, non-responders must be carefully evaluated and withdrawn in a timely fashion. For such non-responders, titration upwards after non-response is contraindicated. If signs of adverse events, such as organ ischemia, appear, the dose should be titrated down as rapidly as hemodynamics allow and combined with epinephrine or norepinephrine to maintain arterial pressure.

2. To decrease capillary leakage:

Vasopressin can be used for both children and adults. In single ventricle physiology, venous pressure tends to be higher than in bi-ventricle patients, especially after the Fontan procedure. In these Fontan cases, patients commonly experience relatively high venous pressure, which expedites capillary leakage together with capillary permeability increased by cytokines derived from surgical intervention. The most common manifestation is increased pleural effusion; low-dose vasopressin can be used for such patients to decrease effusion, with the expectation of decreased capillary leakage. However, intravascular hypovolemia can often occur in those patients. Such a combination of excessive vasoconstriction and hypovolemia is likely to result in organ damage; therefore, high-dose vasopressin is contraindicated, and careful monitoring is mandated.

### 6.2. How to Use

Vasopressin is continuously administered intravenously, initiating with a very low dosage of 0.2–0.3 mU/kg/min, although this depends on facility policies. As we do not currently know of reliable predictors of vasopressin response, patients who are chosen to receive vasopressin (as a second-line drug for non-response to norepinephrine or for catecholamine sparing) may also receive it concomitantly with other vasopressors but always at a low starting dose that is titrated up. There are no set demographic or situationally related guidelines for use, so any patient in need of increased arterial pressure or experiencing VPS/cardiogenic shock symptoms is eligible to receive vasopressin. While monitoring blood pressure, systemic vascular resistance, and SvO2, the dosage is titrated to reach the goal range of relevant hemodynamic parameters. As mentioned above, high dosages can be detrimental and may cause ischemic damage in the organs. In our facility, dosages beyond 1 mU/kg/min are seldom used unless a dire situation demands it. Close monitoring is principal, and titration of vasopressin could be considered yet one more art of the skilled and observant clinician.

### 6.3. What to Look for After Use (Clinical Timeline)

In terms of hemodynamic parameters when administering vasopressin, it is important to monitor arterial blood pressure and systemic vascular resistance, as side effects, including organ ischemia, may occur. Because venous oxygen saturation (SvO2) is a very sensitive measure of organ ischemia, SvO2 monitoring is of importance to prevent adverse events and titrate up to an adequate vasopressin dosage. Abrupt SvO2 drops of 10% or more may indicate adverse events, and checks at 30 min to 1 h intervals are sufficient to evaluate dosage effects and response. Prompt evaluation and treatment should be carried out with respect to individualized criteria, such as the presence of cyanosis, co-morbidities, and/or use of other volemic drugs or drugs with osmolal side effects (e.g., mannitol). For responders, low-dose vasopressin is often enough to achieve the target blood pressure. If high dosages are required in critical situations or for non-responders, other methods should also be considered to deal with the causes (e.g., inotropes for low cardiac output or volume adjustment for hypovolemia). Vasopressin as the sole agent for hemodynamic management should be avoided.

## 7. Conclusions

With a long clinical history, vasopressin and its analogues have shown some utility in the management of vascular and cardiac dynamics across various situations such as septic shock, vasoplegic syndrome, and low cardiac output after surgery. However, mixed results and a scarcity of controlled trials have failed to lend statistical power to recommendations of use after CPB, especially in pediatric patients. Future clinical studies on standardization of use, monitoring guidelines, and testing of new analogues will ensure that vasopressin remains a useful tool in the hemodynamic arsenal.

## Figures and Tables

**Table 1:** Reported clinical studies using vasopressin in cardiovascular surgeries. AVP = arginine vasopressin; Vas. = vascular; Oth. = other; CABG = coronary artery bypass graft; RCT = randomized controlled trial; CPB = cardiopulmonary bypass; IU = international units; U = units; Pros. = prospective; PSM = propensity score matching; NE = norepinephrine; NA = not applicable.

Name/Ref.	Year	Type	Admin. Time	AVP Dosage	Concomitant Drug	Cardiac Surgery Type	Use	Patients (n)	
								Vas.	Oth.
Hasija [67]	2010	RCT	From rewarming until independent from vasopressor support	0.03 IU/min	Saline	On-pump CABG	Intraop.	16	16
Papadopoulos [33]	2010	RCT	20 min before CPB and then 4 h after	0.03 IU/min	Saline	On-pump CABG	Intraop.	25	25
Elgebaly [32]	2012	RCT	10 min before separation from CPB, then 1 h after	0.03 IU/min	Saline	On-pump CABG	Intraop.	10	10
Okamoto [68]	2015	RCT	Administered concomitantly with catecholamines	1.8 U/h	Saline	Cardiac surgery with or without CPB	Intraop.	47	45
Porhomayon [69]	2015	PSM	NA	1 U/h	NA	On-pump CABG	Periop.	280	203
Bomberg [70]	2016	Pros.	Postoperative	1.9 U/h	NA	CABG, valve operation, combined operation, redo	Postop. vasoplegia	11	67
Hajjar [31]	2017	RCT	If vasopressor drugs for vasodilatory shock were required within 48 h after CPB weaning	0.12 U/mL	NE	CABG, valve replacement, or repair surgery with CPB	Postop. vasoplegia	149	151
Jahangirifard [71]	2017	RCT	30 min before the end of CPB, then 1 h after	0.03 IU/min	Saline	Elective CABG with CPB	Intraop.	40	40
Cheng [72]	2018	PSM	Postoperative	0.02–0.07 IU/min	NE	CABG, valve operation	Postop. vasoplegia	169	169
Verma [73]	2022	RCT	During LIMA extraction to end of surgery	0.03 IU/min	Saline	Off-pump CABG	Intraop.	30	30

**Table 2:** Length of stay, vasoplegic syndrome outcomes, and short-term mortality after vasopressin use in cardiovascular surgeries. ICU = intensive care unit; IU = international units; U = units; RCT = randomized controlled trial; Pros. = prospective; PSM = propensity score matching; NE = norepinephrine; NA = not applicable.

Name/Ref.	Year	Study Type	Vas. Dosage	Conco. Drug	Length of Hospital Stay (Days)		Length of ICU Stay (Days)		Vasoplegic Syndrome (%)		Short-term Mortality (%)	
					Vaso.	Oth.	Vas.	Oth.	Vas.	Oth.	Vas.	Oth.
Hasija [67]	2010	RCT	0.03 IU/min	Saline	5.2	5.7	2.1	2.6	NA	NA	0	0
Papadopoulos [33]	2010	RCT	0.03 IU/min	Saline	NA	NA	NA	NA	8	20	0	12
Elgebaly [32]	2012	RCT	0.03 IU/min	Saline	10.7	12.2	5.5	5.9	NA	NA	NA	NA
Okamoto [68]	2015	RCT	1.8 U/h	Saline	8	9	3	3	10.6	20	0	0
Porhomayon [69]	2015	PSM	1 U/h	NA	6.9	6.5	3.4	3.1	NA	NA	1.1	1.5
Bomberg [70]	2016	Pros.	1.9 U/h	NA	35	20	26	9	NA	NA	0	25
Hajjar [31]	2017	RCT	0.12 U/mL	NE	10	13	5	6	NA	NA	0.322	0.49
Jahangirifard [71]	2017	RCT	0.03 IU/min	Saline	NA	NA	3.22	3.22	27.5	30	0	0
Cheng [72]	2018	PSM	0.02–0.07 IU/min	NE	23	24	6	5	NA	NA	5.92	1.77
Verma [73]	2022	RCT	0.03 IU/min	Saline	NA	NA	NA	NA	NA	NA	NA	NA

## Data Availability

No new data were created.

## Abbreviations

**Abbreviation**	**Definition**
ACE	angiotensin-converting enzyme
AVP	arginine vasopressin
BMI	body mass index
CABG	coronary artery bypass graft
CPB	cardiopulmonary bypass
MAP	mean arterial pressure
NO	nitric oxide
NOR	norepinephrine
SvO2	venous oxygen saturation
SVR	systemic vascular resistance
VPS	vasoplegic shock
